# Can adjuvant radiotherapy be omitted for oral cavity cancer patients who received neoadjuvant therapy and surgery? A retrospective cohort study

**DOI:** 10.1097/JS9.0000000000000353

**Published:** 2023-03-31

**Authors:** Wutong Ju, Yiyi Zhang, Ying Liu, Jingjing Sun, Jiang Li, Minjun Dong, Qi Sun, Wentao Shi, Tongchao Zhao, Zhihang Zhou, Yingying Huang, Xinyu Zhou, Dongwang Zhu, Shengjin Dou, Zhiyuan Zhang, Yue He, Chenping Zhang, Ronghui Xia, Guopei Zhu, Laiping Zhong

**Affiliations:** Departments of a Oral and Maxillofacial-Head and Neck Oncology; b Oral Pathology; c Radiology; d Biostatistics Office of Clinical Research Unit, Shanghai Ninth People’s Hospital, Shanghai Jiao Tong University School of Medicine, College of Stomatology, Shanghai Jiao Tong University; e National Center for Stomatology; f National Clinical Research Center for Oral Diseases; g Shanghai Key Laboratory of Stomatology; h Shanghai Research Institute of Stomatology, Shanghai, People’s Republic of China

**Keywords:** adjuvant radiotherapy, de-escalation, neoadjuvant therapy, oral cancer

## Abstract

Surgery and postoperative adjuvant therapy comprise the standard treatment for locally advanced resectable oral squamous cell carcinoma (LAROSCC), while preoperative neoadjuvant therapy is being explored without sufficient confirmation of improved survival. De-escalation regimens after neoadjuvant therapy, such as those omitting adjuvant radiotherapy, may provide comparable or better outcomes, suggesting rigorous assessment of adjuvant therapy outcomes is needed in LAROSCC patients. The authors thus performed this retrospective study in LAROSCC patients who received neoadjuvant therapy and surgery, to compare the outcomes for overall survival (OS) and locoregional recurrence-free survival (LRFS) between the adjuvant radiotherapy (radio) and nonradiotherapy (nonradio) cohorts.

**Materials and methods:**

Patients diagnosed with LAROSCC who received neoadjuvant therapy and surgery were enrolled and divided into radio and nonradio cohorts to determine whether adjuvant radiotherapy could be omitted after neoadjuvant therapy and surgery.

**Results:**

From 2008 to 2021, 192 patients were enrolled. No significant differences were found in OS or LRFS between the radio and nonradio patient cohorts. The 10-year estimated OS rates were 58.9 versus 44.1% in radio versus nonradio cohorts, while 10-year estimated LRFS rates were 55.4 versus 48.2%, respectively. For clinical stage III patients, 10-year OS rates were 62.3 versus 62.6% (radio vs. nonradio), and estimated 10-year LRFS rates were 56.5 versus 60.7% (radio vs. nonradio). Multivariate Cox regression modeling of postoperative variables showed pathologic response of primary tumor and pathologic regional lymph nodes staging were associated with survival, while the adjuvant radiotherapy exposure was not included in the model due to nonsignificance.

**Conclusion:**

These findings support further prospective evaluation of adjuvant radiotherapy omission, and suggest that de-escalation trials are warranted for LAROSCC surgery patients who received neoadjuvant therapy.

## Introduction

Highlights

- No significant differences in survival were found between oral cancer patient cohorts who did or did not receive adjuvant radiotherapy.
- Major pathologic response of neoadjuvant therapy was identified as an independent prognostic indicator.
- Eliminating adjuvant radiotherapy after neoadjuvant therapy and surgery may be feasible.

Oral and pharyngeal squamous cell carcinoma is the eighth most common cancer worldwide^1^. For patients with locally advanced resectable oral squamous cell carcinoma (LAROSCC), surgery followed by adjuvant (chemo)radiotherapy is the standard of care, although clinical outcomes remain relatively poor [i.e. ~50% 5-year overall survival (OS)]^2^,^3^. In addition, due to the complexity of the oral cavity and maxillofacial anatomical structures, surgery for LAROSCC can result in significant functional impairment, potentially leading to serious negative impacts on patient quality of life (QOL). Moreover, adjuvant radiotherapy can cause severe oral mucositis, adversely affect mouth opening, swallowing, and further contribute to other disparaging functional consequences^4^,^5^.

Preoperative neoadjuvant approaches were initially predicted to improve clinical outcomes and increase the likelihood of organ preservation for LAROSCC patients, but previous trials and studies have unfortunately shown no obvious effects of neoadjuvant therapy on survival in LAROSCC^6^,^7^. While more effective neoadjuvant therapeutic approaches for LAROSCC, such as neoadjuvant immunotherapy, targeted therapy, or chemoimmunotherapy combinations are still under exploration^8^, alternative strategies of reducing treatment intensity following neoadjuvant therapy are already showing promising results in clinical trials. For example, a phase II trial reported that neoadjuvant chemotherapy appears to be a feasible treatment option for mandibular preservation in a select group of LAROSCC patients without compromising survival^9^. Similarly, a recent cohort study found that a de-escalation strategy for human papillomavirus (HPV)-associated oropharyngeal cancer treated with definitive chemoradiotherapy showed favorable clinical outcomes and QOL profiles^10^.

For a select set of suitable patients, de-escalating treatment modulation can generate major benefits, such as reducing treatment burden and toxicity, while alleviating healthcare-related financial distress^11^.

Here, we conducted a retrospective study of LAROSCC patients with neoadjuvant therapy and surgery to evaluate the survival of patients who did or did not receive adjuvant radiotherapy. We also discussed whether eliminating adjuvant radiotherapy may be appropriate for these patients.

## Materials and methods

### Patients and treatment

This retrospective study was performed at our center. Data from LAROSCC patients who received neoadjuvant therapy and surgery at our department from 2008 to 2021 were collected. This study followed the ethical guidelines of the Declaration of Helsinki and was approved by the Institutional Ethics Committee of our center (registration number NCT05455632, clinicaltrials.gov). Signed informed consent was obtained from each enrolled patient. This work has been reported in line with the STROCSS criteria^12^, Supplemental Digital Content 1, http://links.lww.com/JS9/A205.

Inclusion criteria included: age 18–75; male or female; histopathologic diagnosis of primary oral squamous cell carcinoma (including sites of the tongue, gums, bucca, floor of the mouth, hard palate, and posterior molar region); and clinical stage of III/IVA (cT1-2/cN1-2/M0 or cT3-4a/cN0-2/M0, American Joint Committee on Cancer 8th edition).

Exclusion criteria included: patients who received radiotherapy targeting the head and neck region or neck dissection for any other disease prior to our treatment; evidence of recurrence or metastasis was assessed by physical examination reports or imaging studies (i.e. ultrasonography, computed tomography, positron emission tomography-computed tomography, or MRI) before initiation of adjuvant radiotherapy; evidence of serious and/or unrelieved side effects of neoadjuvant therapy or postoperative complications that resulted in failure to receive adjuvant radiotherapy.

Surgery and adjuvant radiotherapy were conducted according to the National Comprehensive Cancer Network (NCCN) guidelines for oral cavity cancer. Patients were followed-up with physical examination and computed tomography or ultrasonography imaging every 3 months for years 1–2, then every 6 months for years 3–5, and every 12 months for years 5–10.

### Neoadjuvant therapy response analysis

Primary specimens underwent a standard pathologic evaluation by two certified dermatopathologists. Response to neoadjuvant therapy was assessed by thorough examination of tumor sections after surgery, as previously reported^6^,^13^,^14^. The residual viable tumor cell percentage was evaluated using resected tumor slides. Major pathologic response (MPR) was defined as the presence of 10% or fewer residual viable tumor cells. Pathologists were blinded to patient grouping.

### Statistical analysis

The Kaplan–Meier method was used to calculate OS rates (time elapsed from the date of surgery to date of death or censoring at the last follow-up) and locoregional recurrence-free survival rates (LRFS; the time elapsed from the date of surgery to the first signs or symptoms of locoregional recurrence). Log-rank tests were used to assess differences in OS and LRFS between groups, while the Cox proportional hazards regression model was used to compare factors with prognostic potential. Correlations between survival and each covariable were examined via a univariable Cox proportional hazards regression model followed by a preliminary multivariable Cox proportional hazards regression model. Propensity score matching was implemented to regulate potential bias of sampling. The variables included in the propensity score matching were age, sex, clinical T and N stage. To maximize data utilization, we used caliper matching with a match tolerance with 0.02. *P* less than 0.05 was defined as significant. The significance level for two-sided *P* values was set at 0.05 in statistical analyses. Statistical analysis was performed in IBM SPSS Statistics (version 23) and GraphPad Prism software (version 9.1.2).

## Results

### Patient information

**Figure 1:**
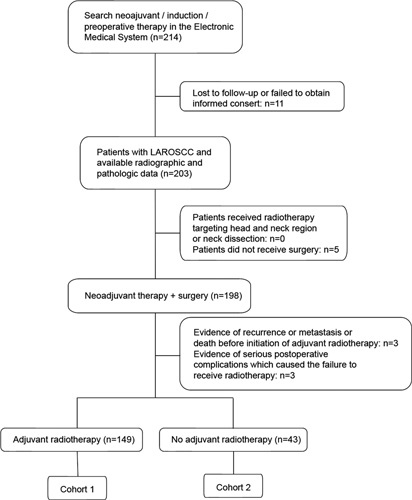
Study flow. LAROSCC, locally advanced resectable oral squamous cell carcinoma.

Two hundred three (203) patients who received neoadjuvant therapy and surgery for LAROSCC at our department from 2008 to 2021 were screened, among whom 149 patients (77.6%) underwent neoadjuvant therapy, surgery, and adjuvant radiotherapy and were included in cohort 1 (radio cohort). Forty-three (43) patients (32.4%) who underwent neoadjuvant therapy and surgery without adjuvant radiotherapy were included in cohort 2 (nonradio cohort). Neoadjuvant therapy approaches included: Docetaxel plus cisplatin plus fluorouracil (TPF) regimen (*n*=140, 72.9%); docetaxel plus cisplatin plus cetuximab (TPE) regimen (*n*=33,17.2%); or PD-1 inhibitor plus VEGFR inhibitor regimen (*n*=19, 9.8%). Total radiation doses ranged from 50 to 70 Gy in the radio cohort (Fig. 1 for a flowchart of the cohort selection process).

Patient characteristics and demographics in each cohort are compared and listed in Table 1. Clinical stage IV was more common in the nonradio cohort (55.8 vs. 36.2%, *P*=0.034). No positive or close margin was found for all enrolled patients. Three patients (3/149, 2.0%) in the radio cohort received adjuvant chemotherapy, whereas one (1/43, 2.3%) did in the nonradio cohort (*P*>0.05).

**Table 1:** Patient demographic and characteristics.

		Cohort		
Characteristics	Total patients (*N*=192) [*N* (%)]	Radio [*n* (%)]	Nonradio [*n* (%)]	*P* ^a^
Sex				
Male	134 (69.8)	102 (68.5)	32 (74.4)	0.572
Female	58 (30.2)	47 (31.5)	11 (25.6)	
Age (years)				
<60	122 (63.5)	96 (64.4)	26 (60.5)	0.720
≥60	70 (36.5)	53 (35.6)	17 (39.5)	
Clinical T stage				
T1	3 (1.6)	2 (1.3)	1 (2.3)	0.003
T2	46 (24)	36 (24.2)	10 (23.3)	
T3	90 (46.9)	79 (53)	11 (25.6)	
T4	53 (27.6)	32 (21.5)	21 (48.8)	
Clinical N stage				
N0	71 (37)	60 (40.3)	11 (25.6)	0.187
N1	80 (41.7)	58 (38.9)	22 (51.2)	
N2	41 (21.4)	31 (20.8)	10 (23.3)	
Clinical stage				
III	114 (59.4)	95 (63.8)	19 (44.2)	0.034
IVA	78 (40.6)	54 (36.2)	24 (55.8)	
Pathologic stage				
I	16 (8.3)	14 (9.4)	2 (4.7)	<0.001
II	2 (1.0)	2 (1.3)	0	
III	85 (44.3)	61 (40.9)	24 (55.8)	
IVA	89 (46.4)	72 (48.3)	17 (39.5)	
Down-stage^b^				
Yes	33	23	10	0.254
No	159	126	33	
Pathologic response^c^				
MPR	54 (28.1)	40 (74.0)	14 (25.9)	0.311
Non-MPR	135 (70.3)	109 (80.7)	26 (19.3)	
Smoking status^d^				
Current/former	88 (45.8)	71 (47.7)	17 (39.5)	0.388
Never	104 (54.2)	78 (52.3)	26 (60.5)	
Alcohol use^e^				
Positive	74 (38.5)	59 (39.6)	15 (34.9)	0.599
Negative	118 (61.5)	90 (60.4)	28 (65.1)	
Nerve invasion				
No nerve invasion	165 (85.9)	128 (77.6)	37 (22.4)	0.981
Nerve invasion	27 (14.1)	21 (77.8)	6 (22.2)	
Lymphovascular invasion				
Yes	5	5	0	0.589
No	187	144	43	

^a^ *P* value from *χ* ^2^-test was reported to compare baseline characteristics between the two cohorts.

^b^ Down-stage was defined as down staging from baseline clinical stage to pathologic stage.

^c^ MPR (major pathological response) was defined as less than or equal to 10% residual viable tumor cells.

^d^ Former/current smokers defined as at least a one pack-year history of smoking.

^e^ Positive alcohol use was defined as current alcohol use of more than one drink per day for 1 year (12 ounces of beer with 5% alcohol, or 5 ounces of wine with 12–15% alcohol, or one ounce of liquor with 45–60% alcohol). All other patients were classified as negative alcohol use.

### Survival analysis

**Figure 2:**
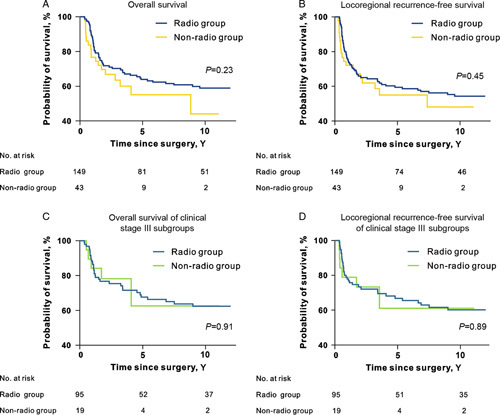
Survival curves of radio and nonradio cohorts. (A) Overall survival curves of two cohorts. (B) Locoregional recurrence-free survival curves of two cohorts. (C) Overall survival curves of clinical stage III subgroups. (D) Locoregional recurrence-free survival curves of clinical stage III subgroups. Kaplan–Meier method was used for survival analysis. Log-rank *P* values were presented in the figure.

As of December 2021, the estimated 10-year OS rates were 58.9 versus 44.1% (radio versus nonradio, *P*=0.23; Fig. 2A) and the estimated 10-year LRFS rates were 54.4 versus 48.2% (radio vs. nonradio, *P*=0.45; Fig. 2B), with a median follow-up time of 49 months (range: 15–144 months).

Since clinical stage IV was more common in the nonradio cohort, we performed separate subgroup survival analyses for clinical stage III and stage IV patients. Among patients with clinical stage IV diseases, the 10-year OS rates were 52.6 versus 32.7% (radio vs. nonradio, *P*=0.20; Supplementary Fig. 1A, Supplemental Digital Content 2, http://links.lww.com/JS9/A206), while the estimated 10-year LRFS rate was 56.5 versus 60.7% (radio versus nonradio, *P*=0.72; Supplementary Fig. 1B, Supplemental Digital Content 2, http://links.lww.com/JS9/A206). Interestingly, survival rates of clinical stage III patients were relatively close between the radio and nonradio cohorts: 10-year OS rates of 62.3 versus 62.6% (radio vs. nonradio, *P*=0.91; Fig. 2C) and estimated 10-year LRFS rates of 56.5 versus 60.7% (radio vs. nonradio, *P*=0.89; Fig. 2D).

Univariable and multivariate analyses were conducted using baseline variables, including age, sex, clinical primary tumor (cT) stage, clinical regional lymph nodes (cN) stage, cohort (radio vs. nonradio), and adjuvant chemotherapy (adjuvant chemotherapy vs. nonadjuvant chemotherapy). Multivariate Cox regression model 1 for baseline variables revealed that cN stage was independently associated with both OS and LRFS, while no significant differences were found between the radio and nonradio cohorts (Table 2; Supplementary Table 1, Supplemental Digital Content 2, http://links.lww.com/JS9/A206).

**Table 2:** Cox regression analysis of baseline variables from two cohorts for overall survival.

	Univariable analysis		Multivariable analysis	
Characteristics	HR (95% CI)	*P* ^a^	HR (95% CI)	*P* ^a^
Sex (male vs. female)	1.106 (0.673–1.819)	0.691		
Age (≥60 vs. <60)	1.224 (0.769–1.950)	0.394		
Clinical T stage (≥T3 vs. <T3)	0.848 (0.516–1.395)	0.516	1.210 (0.709–2.063)	0.485
Clinical N stage (N+ vs. N0)	2.102 (1.237–3.572)	0.006	2.233 (1.262–3.952)	0.006
Group (nonradio vs. radio)	1.382 (0.810–2.359)	0.235	1.289 (0.754–2.205)	0.354
Adjuvant chemotherapy (yes vs. non)	2.391 (0.751–7.610)	0.140		
Smoking status (current/former vs. never)	1.254 (0.797–1.972)	0.327		
Alcohol use (positive vs. negative)	1.031 (0.650–1.637)	0.896		

^a^ *P* values from univariable and multivariable Cox regression models. HR, hazard ratio

Given the significant disparity in sample size between the two cohorts, we used propensity score matching to control for potential selection bias, which resulted in a subset of 82 patients (41 per cohort) matched for age, sex, cT, and cN stage (match tolerance=0.02). No significant differences were detected in either OS or LRFS between the matched cohorts (Supplementary Fig. 2, Supplemental Digital Content 2, http://links.lww.com/JS9/A206).

**Figure 3:**
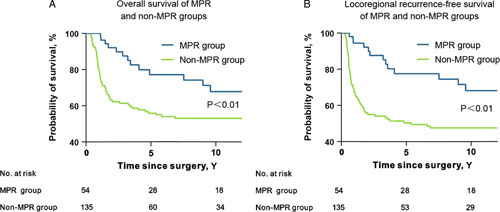
Survival curves of major pathologic response (MPR) and non-MPR groups. (A) Overall survival and (B) locoregional recurrence-free survival of MPR and non-MPR groups. Kaplan–Meier method was used for survival analysis. Log-rank *P* values were presented in the figure.

Since MPR is currently considered the most reliable prognostic predictor for patients who received neoadjuvant therapy and can only be obtained after surgery^15^,^16^, we compared OS and LRFS between MPR and non-MPR groups, and repeated univariable and multivariate analyses using postoperative variables, including age, sex, pathologic regional lymph nodes (pN) stage, cohort (radio vs. nonradio), adjuvant chemotherapy (adjuvant chemotherapy vs. nonadjuvant chemotherapy), and pathologic response (MPR vs. non-MPR). The pathologic primary tumor (pT) stage variable was not included in the multivariate analysis because it was significantly affected by pathologic response. Evaluations of pathologic response were available for 98.4% (189/192) cases, and 54 cases showed MPR while 135 cases were non-MPR. The estimated 10-year OS rates were 67.9 versus 53.1% (MPR vs. non-MPR, *P*<0.01; Fig. 3A); estimated 10-year LRFS rates were 68.2% versus 47.6% (MPR vs. non-MPR, *P*<0.01; Fig. 3B). Multivariate Cox regression model 2 for postoperative variables showed that pathologic response was independently associated with OS and LRFS, while differences between radio and nonradio cohorts were not significant and therefore excluded from the model (Table 3, Supplementary Table 2, Supplemental Digital Content 2, http://links.lww.com/JS9/A206).

**Table 3:** Cox regression analysis of postoperative variables from two cohorts for overall survival.

	Univariable analysis		Multivariable analysis	
Characteristics	HR (95% CI)	*P* ^a^	HR (95% CI)	*P* ^a^
Sex (male vs. female)	1.106 (0.673–1.819)	0.691		
Age (≥60 vs. <60)	1.224 (0.769–1.950)	0.394		
Pathologic T stage				
T1	1	<0.001		
T2	1.232 (0.517–2.938)	0.638		
T3	1.361 (0.624–2.971)	0.438		
T4	3.676 (1.639–8.245)	0.002		
Pathologic N stage				
N0	1	0.001	1	0.002
N1	1.047 (0.487–2.253)	0.906	1.091 (0.502–2.372)	0.826
N2	2.296 (1.377–23.830)	0.001	2.445 (1.401–4.266)	0.002
N3	5.140 (1.759–15.013)	0.003	3.604 (1.118–11.625)	0.032
Group (nonradio vs. radio)	1.382 (0.810–2.359)	0.235	1.674 (0.860–3.258)	0.129
Adjuvant chemotherapy (yes vs. non)	2.391 (0.751–7.610)	0.140		
Smoking status (current/former vs. never)	1.254 (0.797–1.972)	0.327		
Alcohol use (positive vs. negative)	1.031 (0.650–1.637)	0.896		
Pathologic response^b^ (non-MPR vs. MPR)	2.254 (1.237–4.107)	0.008	2.024 (1.101–3.722)	0.023
Nerve invasion (invasion vs. noninvasion)	1.009 (0.519–1.965)	0.978		

^a^ *P* values from univariable and multivariable Cox regression models.

^b^ MPR (major pathological response) was defined as less than or equal to 10% residual viable tumor cells.

## Discussion

Among LAROSCC patients who received neoadjuvant therapy and surgery, survival rates were found not significantly lower for the nonradio cohort than for the radio cohort. These findings provide initial evidence supporting that LAROSCC patients who receive neoadjuvant therapy and surgery do not appear to benefit from adjuvant radiotherapy, which is in general agreement with results reported by Dhere *et al.* 17.

In our analysis, the baseline Cox regression model 1 showed no direct correlation between baseline cT stage with OS or LRFS in these patients. By contrast, baseline late cN stage emerged as an independent survival risk factor, despite intensive therapeutic interventions, including neoadjuvant therapy, surgery of primary tumor, necessary neck dissection, with or without adjuvant radiotherapy. These results suggest that further studies are warranted assessing the use of neoadjuvant therapies in patients with late baseline cN stage.

Clinical trials are underway to investigate de-escalation treatments in patients with HPV-associated head and neck cancer and are maintaining excellent outcomes^18^. For it is now accepted that HPV-positive and HPV-negative head and neck cancers are distinct diseases and oral cavity cancer are mostly identified as HPV-negative^19^. Adjuvant radiotherapy is still necessary for LAROSCC patients who received upfront surgery according to guidelines and Awan’s research, which suggested that eliminating adjuvant radiotherapy is inappropriate in many pathologic stage III or IVa patients^20^. However, in the current study of LAROSCC patients with neoadjuvant therapy, MPR was further demonstrated to serve as a reliable predictor of better survival. In addition, the postoperative Cox regression model 2 implied that de-escalation approaches, such as those omitting adjuvant therapy, are potentially suitable for MPR patients with early pN stage. This opinion aligns well with findings of a phase II trial that show patients with pN0 head and neck squamous cell carcinoma may not benefit from adjuvant radiotherapy, based on excellent control rates in unirradiated neck tissue and the absence of long-term adverse impacts on QOL^21^. Another case-match multicenter study showed adjuvant radiotherapy might not be necessary for LAROSCC patients treated with appropriate surgery^22^, which further indicates that de-escalation trials for adjuvant therapy could be feasible. In our study, we aimed to provide a concept of using neoadjuvant therapy as a substitute and to screen out patients with MPR to receive de-escalation therapy, and finally improve patients’ tumor control rate and QOL.

This study was limited by relatively small sample size and large disparity in patient number between the radio and nonradio cohorts, which could introduce bias and restrict the statistical power of conclusions based on these analyses. However, the absence of positive benefits of adjuvant therapy, which support its omission from future treatment regimens, was also demonstrated through propensity score matching. This internal validation method was selected over other approaches because matching techniques have been shown to produce stable and unbiased estimates of predictive accuracy with increased power and decreased variability, regardless of the sample size. In addition, data in this study were collected retrospectively with varying follow-up times and heterogeneous neoadjuvant therapies. Future work will include a large cohort from multiple centers to compare patient cohorts with uniform interventions and longer follow-up times, which could inform the applicability of using MPR as a prognostic predictor and a potential indicator for de-escalation trials.

In conclusion, for LAROSCC patients who received neoadjuvant therapy and surgery, pN0 and MPR predict significant better survival, while no statistical differences are detected in OS and LRFS between patient cohorts with or without adjuvant radiotherapy. The findings support further prospective evaluation of de-escalation trials for LAROSCC surgery patients who received neoadjuvant therapy.

## Ethical approval

This study followed the ethical guidelines of the Declaration of Helsinki and was approved by the Institutional Ethics Committee, Ninth People’s Hospital, Shanghai Jiao Tong University School of Medicine. This study was registered at clinicaltrials.gov (registration number: NCT05455632, Hyperlink: https://clinicaltrials.gov/ct2/show/NCT05455632?term=NCT05455632&draw=2&rank=1).

## Sources of funding

This work was supported by the National Natural Science Foundation of China (Grant Numbers 81972525, 82172734, 82103043) and the Science and Technology Commission of Shanghai Municipality (Grant Number 21Y21900300).

## Author contribution

R.X., G.Z., L.Z.: conceptualization. W.J., Y.Z., Y.L., J.S., J.L., M.D., Q.S., T.Z., Z.Z., Y.H., X.Z., D.Z., S.D.: investigation. W.J., Y.Z., W.S.: data curation, formal analysis, methodology, roles/writing – original draft. W.J., L.Z.: funding acquisition. Z.Z., Y.H., C.Z., L.Z.: writing – review and editing, supervision. All authors read and approved the final manuscript.

## Conflicts of interest disclosure

All authors have no conflicts of interest to declare.

## Guarantor

Laiping Zhong, Department of Oral and Maxillofacial-Head and Neck Oncology, Shanghai Ninth People’s Hospital, College of Stomatology, Shanghai Jiao Tong University School of Medicine, No. 639 Zhizaoju Road, Shanghai 200011, P.R. China. Tel: +86 212 327 1699 5156. E-mail: zhonglp@hotmail.com

## Data statement

Most de-identified clinical data of individual patients are available in the manuscript or additional files. Other data can be obtained upon scientifically sound request from the corresponding author at: zhonglp@hotmail.com

## Provenance and peer review

Not commissioned, externally peer-reviewed.

## Supplementary Material
