# Supplementary material for: Can adjuvant radiotherapy be omitted for oral cavity cancer patients who received neoadjuvant therapy and surgery? A retrospective cohort study
Source: Int J Surg. 2023 Mar 31;109(4):879–86. doi: 10.1097/JS9.0000000000000353 (PMC10389426; doi:10.1097/JS9.0000000000000353)
Supplement: Supplementary file 2 [file js9-109-0879-s002.doc]

**Supplementary table 1.** Cox regression analysis of baseline variables from two cohorts for locoregional recurrence-free survival.

| Characteristics | Univariable analysis | | Multivariable analysis | |
| --- | --- | --- | --- | --- |
|  | HR (95% CI) | *P* | HR (95% CI) | *P* |
| Sex (male vs female) | 1.506 (0.660-1.687) | 0.821 |  |  |
| Age (≥60 vs <60) | 1.241 (0.799-1.926) | 0.337 |  |  |
| Clinical T stage  (≧T3 vs＜T3) | 0.839 (0.522-1.349) | 0.469 | 1.213 (0.729-2.018) | 0.457 |
| Clinical N stage  (N+ vs N0) | 2.143 (1.295-3.545) | 0.003 | 2.296 (1.336-3.945) | 0.003 |
| Group  (non-radio vs radio) | 1.217 (0.727-2.037) | 0.455 | 1.127 (0.672-1.891) | 0.650 |
| Adjuvant chemotherapy  (yes vs non) | 2.040 (0.642-6.477) | 0.227 |  |  |
| Smoking Status**  (current/former vs never) | 1.260 (0.819-1.938) | 0.292 |  |  |
| Alcohol Use***  （positive vs negative） | 1.104 (0.713-1.710) | 0.658 |  |  |

* *P* value from univariable and multivariable cox regression were reported to estimate the survival rate of the event endpoint

**Former/current smokers defined as at least a one pack-year history of smoking.

***Positive alcohol use was defined as current alcohol use of more than one drink per day for 1 year (12 ounces of beer with 5% alcohol, or 5 ounces of wine with 12%-15% alcohol, or one ounce of liquor with 45%-60% alcohol). All other patients were classified as negative alcohol use.

**Supplementary table 2.** Cox regression analysis of postoperative variables from two cohorts for locoregional recurrence-free survival.

| Characteristics | Univariable analysis | | Multivariable analysis | |
| --- | --- | --- | --- | --- |
|  | HR (95% CI) | *P** | HR (95% CI) | *P** |
| Sex (male vs female) | 1.506 (0.660-1.687) | 0.821 |  |  |
| Age (≥60 vs <60) | 1.241 (0.799-1.926) | 0.337 |  |  |
| Pathologic T stage | | | | |
| T1 | 1 | <0.001 |  |  |
| T2 | 1.439 (0.615-3.364) | 0.401 |  |  |
| T3 | 1.542 (0.713-3.334) | 0.271 |  |  |
| T4 | 4.179 (1.875-9.315) | <0.001 |  |  |
| Pathologic N stage | | | | |
| N0 | 1 | <0.001 | 1 | 0.001 |
| N1 | 1.339 (0.676-2.656) | 0.403 | 1.383 (0.691-2.768) | 0.360 |
| N2 | 2.223 (1.356-3.645) | 0.002 | 2.140 (1.259-3.628) | 0.005 |
| N3 | 6.615 (2.506-17.461) | <0.001 | 5.937 (1.995-17.672) | 0.001 |
| Group  (non-radio vs radio) | 1.217 (0.727-2.037) | 0.455 | 1.267 (0.660-2.432) | 0.478 |
| Adjuvant chemotherapy  (yes vs non) | 2.040 (0.642-6.477) | 0.227 |  |  |
| Smoking Status**  (current/former vs never) | 1.260 (0.819-1.938) | 0.292 |  |  |
| Alcohol Use***  （positive vs negative） | 1.104 (0.713-1.710) | 0.658 |  |  |
| Pathologic Response****  (non-MPR vs MPR) | 2.719 (1.501-4.925) | 0.001 | 2.489 (1.363-4.545) | 0.003 |
| Nerve Invasion  (invasion vs non-invasion) | 1.308 (0.723-2.365) | 0.375 |  |  |

* *P* value from univariable and multivariable cox regression were reported to estimate the survival rate of the event endpoint

**Former/current smokers defined as at least a one pack-year history of smoking.

***Positive alcohol use was defined as current alcohol use of more than one drink per day for 1 year (12 ounces of beer with 5% alcohol, or 5 ounces of wine with 12%-15% alcohol, or one ounce of liquor with 45%-60% alcohol). All other patients were classified as negative alcohol use.

****MPR (Major pathological response) was defined as ≤10% residual viable tumor cells.


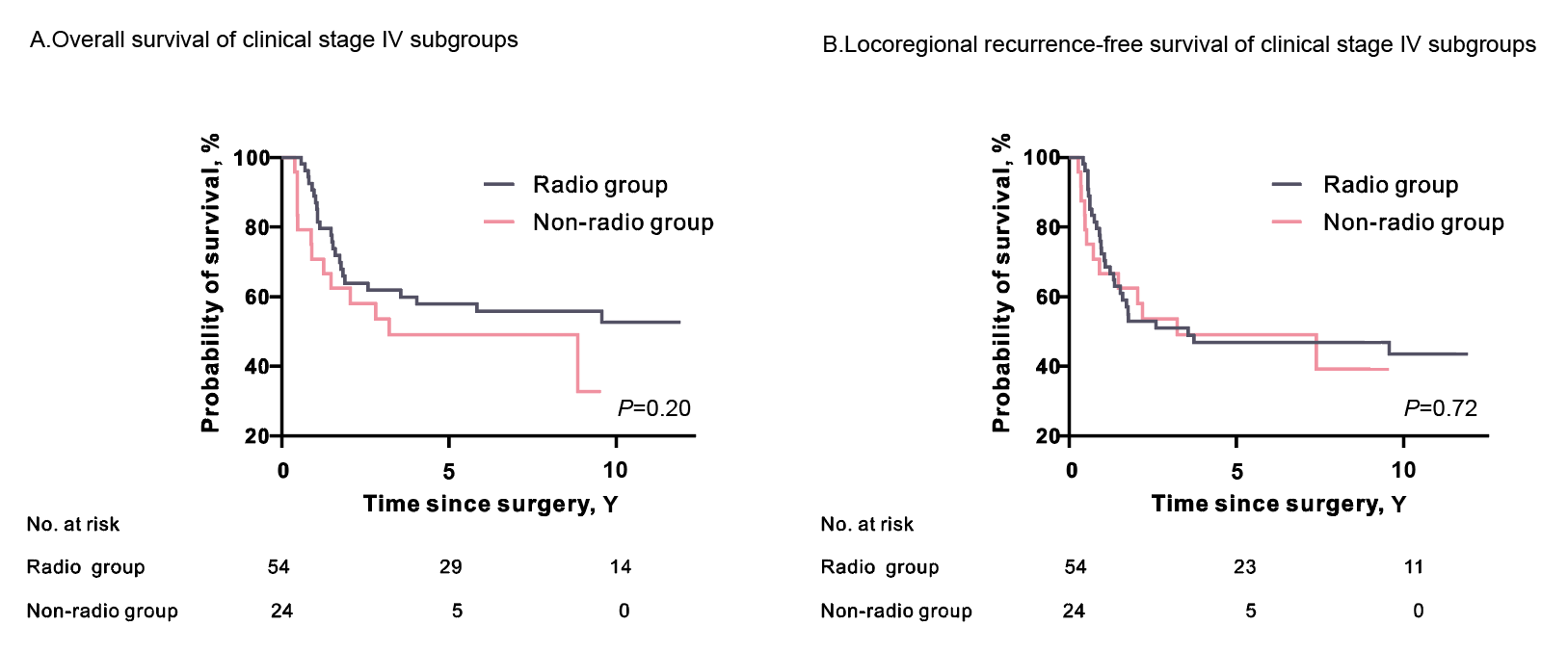


**Supplementary Fig. 1 Survival curves of clinical stage IV subgroups.** (A) Overall survival and (B) locoregional recurrence free survival of clinical stage IV subgroups. Kaplan-Meier method was used for survival analysis, Log-rank *P* values were presented in the figure.


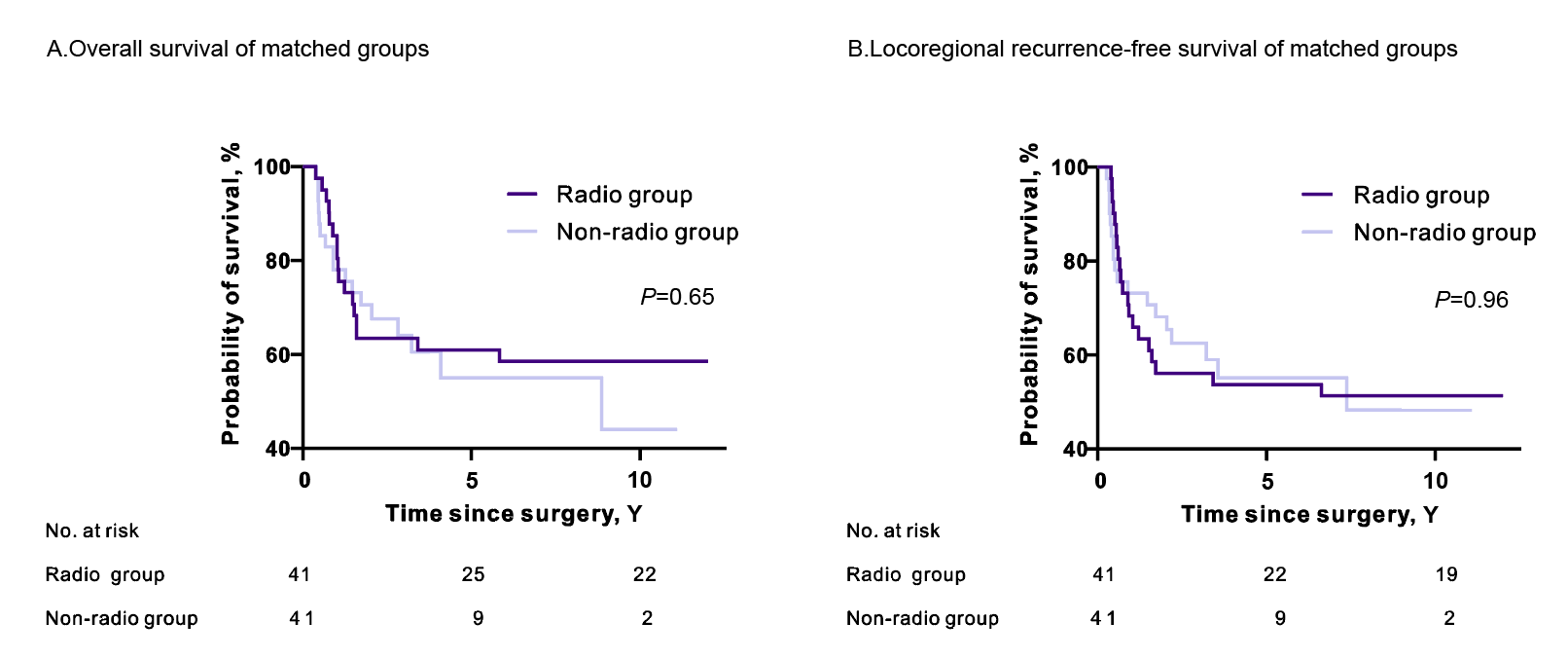


**Supplementary Fig. 2 Survival curves of matched groups.** (A) Overall survival and (B) locoregional recurrence free survival of matched groups. The propensity score matching method was used for matching. Kaplan-Meier method was used for survival analysis, Log-rank *P* values were presented in the figure.
